# Supplementary material for: The causal relationship between the human gut microbiota and pyogenic arthritis: a Mendelian randomization study
Source: Front Cell Infect Microbiol. 2024 Nov 26;14:1452480. doi: 10.3389/fcimb.2024.1452480 (PMC11629706; doi:10.3389/fcimb.2024.1452480)
Supplement: Supplementary file 1 [file DataSheet1.zip › mendelian test/ebi-a-GCST90027531.csv_forest.pdf]

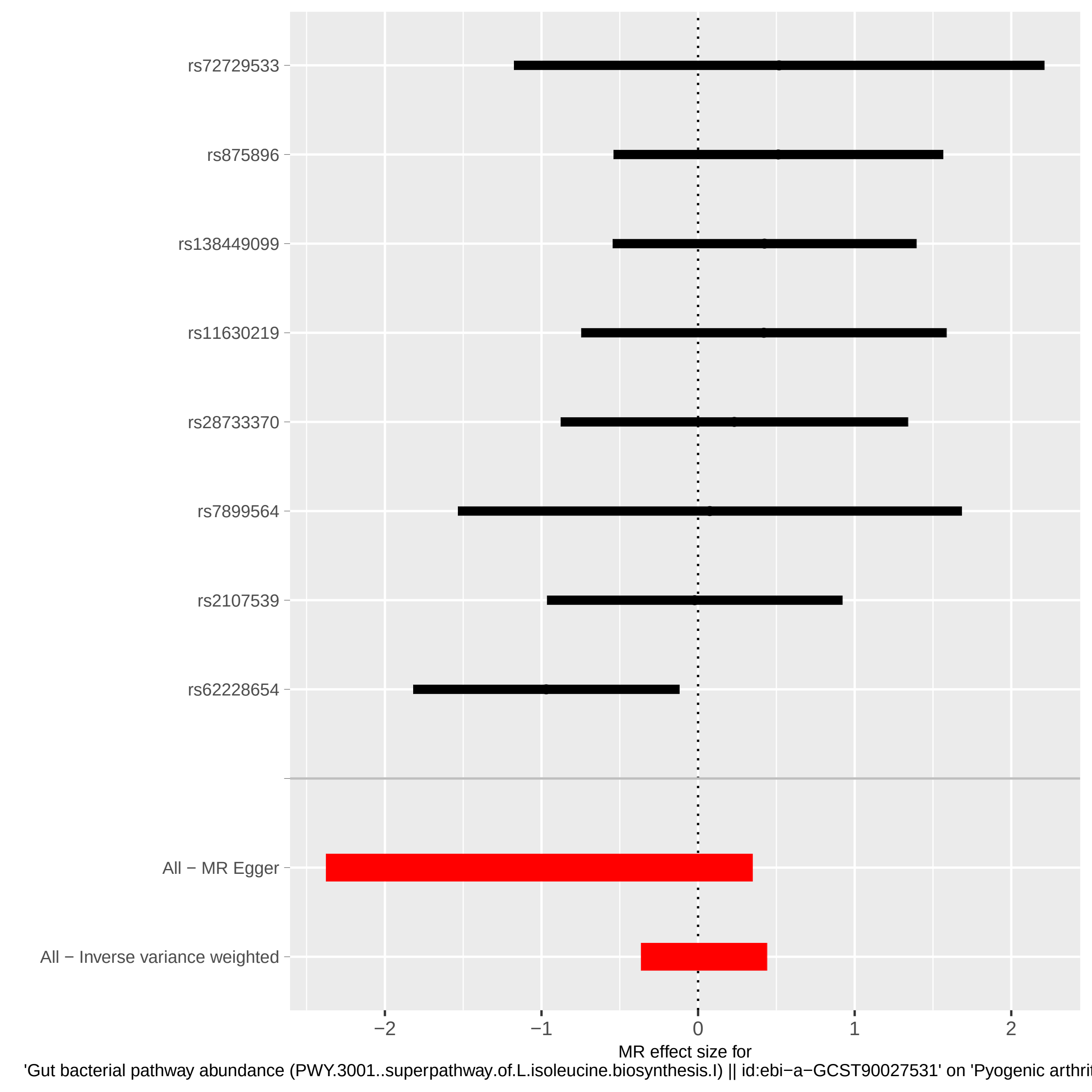
rs72729533
rs875896
rs138449099
rs11630219
rs28733370
rs7899564
rs2107539
rs62228654
All − MR Egger
All − Inverse variance weighted
−2
−1
0
1
2
MR effect size for
'Gut bacterial pathway abundance (PWY.3001..superpathway.of.L.isoleucine.biosynthesis.I) || id:ebi−a−GCST90027531' on 'Pyogenic arthri
